# Taxonomic notes on the genus *Hexanchorus* Sharp, 1882 and *Heterelmis* Sharp, 1882 from Bolivia (Coleoptera, Elmidae)

**DOI:** 10.3897/zookeys.1270.178101

**Published:** 2026-02-19

**Authors:** Marek Linský, Fedor Čiampor

**Affiliations:** 1 Plant Science and Biodiversity Centre, Slovak Academy of Sciences, Dúbravská cesta 9, SK-84523, Bratislava, Slovakia Plant Science and Biodiversity Centre, Slovak Academy of Sciences Bratislava Slovakia

**Keywords:** Larainae, Neotropical region, new species, Philibert Germain, taxonomy

## Abstract

The genus *Hexanchorus* Sharp, 1882 is represented in Bolivia by one species, *H. tibialis* Hinton, 1935. A series of this species was originally collected by Germain in the Yungas of Cochabamba and, in 1935, described by Hinton based on a single specimen that is in poor condition and lacks several key characters. Here, we redescribe *H. tibialis* based on additional material collected by Germain and describe a new species, *H. siriono***sp. nov**., from Santa Cruz, Bolivia, which exhibits striking male sexual dimorphism in the protarsi. Both species are illustrated with photographs of the habitus and drawings of the male genitalia, and for *H. siriono***sp. nov**. also the female genitalia. Germain’s journey in Bolivia and its known elmid fauna are briefly discussed. Additionally, *Helmis cervina* Grouvelle, 1896 and *Helmis longior* Grouvelle, 1896, both from Bolivia, are formally transferred to *Heterelmis* Sharp, 1882. The lectotype of the latter species is designated.

## Introduction

The Elmidae are a large family of water beetles inhabiting lotic environments on all continents except Antarctica. The recent catalogue of the family recognizes over 1,500 species in about 150 genera (Jäch et al. 2016), traditionally divided into two subfamilies: the aquatic Elminae and the mostly semiaquatic Larainae (Kodada et al. 2016). The greatest diversity of Elmidae occurs in montane regions of tropical and subtropical rainforests (Jäch and Balke 2008). The Neotropical realm harbours, with 52 genera, roughly one-third of the global diversity of riffle beetles, six of which were described in the last decade (Barr 2018; Čiampor et al. 2019; González-Córdoba et al. 2021; Linský et al. 2022; Polizei et al. 2022; Barr et al. 2025).

Although the riffle-beetle fauna of several South American countries has been studied to some extent, much of the continent’s biodiversity remains insufficiently explored. Bolivia has received comparatively little attention, largely due to historical, political, and logistical reasons. The first known Bolivian specimens of Elmidae were collected by P. Germain, who visited the country between 1887 and 1889 (Germain 1897, 1900a); these were subsequently examined and characterized by Grouvelle (1896). One specimen from this material was later described by Hinton (1935) as *Hexanchorus tibialis*, the first representative of this genus known from Bolivia. The genus *Hexanchorus* was recently reviewed based on the material from Ecuador and, with the description of *H. siriono* sp. nov., includes 26 known species (Linský et al. 2019, 2022).

Nearly 50 years after Germain’s voyage, additional material was collected by Hinton, who joined the Percy Sladen Trust Expedition to Lake Titicaca in 1937 and served as its entomologist and interpreter (Salt 1978). Despite the primary focus on Lake Titicaca, Hinton was also able to collect near Lake Poopó and La Paz in Bolivia during this time (Gilson 1939; Hinton 1940b). After completing his work around the lake, he continued on to Bolivia, travelling by steamer and rail to La Paz, then by air to Trinidad, and onward through the Mamoré and Madeira rivers to Manaus, Brazil (Gilson 1939). During this trip, he collected additional beetle material, which he described shortly thereafter.

The Bolivian fauna of riffle beetles was last summarized by Manzo and Moya (2010), who recorded 32 taxa in 12 genera. Two species had an unclear position, as they were originally described in the genus *Helmis* Bedel, 1878 (an unjustified emendation of *Elmis* Latreille, 1802), which has a Palaearctic distribution (Jäch et al. 2016). Manzo and Moya noted that the first species, *H. longior* (Grouvelle, 1896), likely belongs to *Heterelmis* Sharp, 1882. In fact, this species had already been validly, though cryptically, transferred to *Heterelmis* by Hinton (1940a: 369–371). It is mentioned in three figures (pp. 369–370), in the text (p. 371), and in the volume index (p. 514). The second species, *H. cervina* (Grouvelle, 1896), comb. nov., is formally transferred to *Heterelmis* in this paper.

Subsequently, Manzo (2013) recorded *Xenelmis leechi* Perkins & Steiner, 1981, as a new genus and species record for Bolivia. Jäch et al. (2016) reported three additional species from the country: *Hexacylloepus nothrus* Spangler, 1966, *Macrelmis isus* (Hinton, 1946), and *M. saltensis* Manzo, 2003. Finally, Polizei and Barclay (2019a, 2019b) reported *Hintonelmis anamariae* Fernandes et al., 2010, also a new generic record for Bolivia, which was previously known from Brazil and Ecuador, and the new species *Cylloepus segurae* Polizei & Barclay, 2019.

## Material and methods

The studied material was loaned from various museum collections. Specimens were dried, pinned on point or on card or fixed in pure alcohol. Morphological characters were examined under a Leica M205C stereomicroscope at magnifications up to 160×. Male and female genitalia were studied as temporary glycerin slides at magnifications up to 600×, using a Leica DM1000 light microscope. Drawings were made with a drawing tube, subsequently scanned and finalized in Adobe Photoshop 2025. Habitus photographs were taken using a Zeiss Axio Zoom.V16 stereomicroscope, diffuse LED lighting, a Canon 5D Mark IV camera, and Zerene Stacker software. The beginning and end of label texts are indicated by double quotation marks (“ ”); a double slash (//) separates the data on different labels; square brackets ([ ]) are used to indicate authors’ comments. Morphological terms generally follow Kodada et al. (2016).

Metric characters were measured with an ocular grid to nearest 0.05 mm. For specimens available only as photographs, measurements were taken from calibrated images provided by the personnel of the Muséum national d’Histoire naturelle, Paris.

Abbreviations used in the text: **CL** – body length (measured from anterior margin of pronotum to elytral apices), **EL** – elytral length, **EW** – maximum elytral width, **PL** – pronotal length, **PW** – maximum pronotal width, **MNHN** – Muséum national d’Histoire naturelle (Paris, France), **NHMUK** – Natural History Museum (London, United Kingdom), **OMNH** – Sam Noble Oklahoma Museum of Natural History (Norman, Oklahoma, USA).

## Results

### Taxonomy

#### Hexanchorus tibialis

Taxon classificationAnimaliaColeopteraElmidae

Hinton, 1935

DC2EFDB1-4DA3-52A5-9DFC-D1D996CE4B78

Figs 1, 2, 5 D, 6A

##### Type material examined.

(1 ♂) ***Holotype***: Bolivia • ♂ “Type // Bolivia // Yungas de Cochabamba Bolivie // Fry Coll. 1905.100. // 69276 // type tibialis H.E. Hinton // NHMUK 010875808” (NHMUK).

##### Condition.

**Figure 1.**
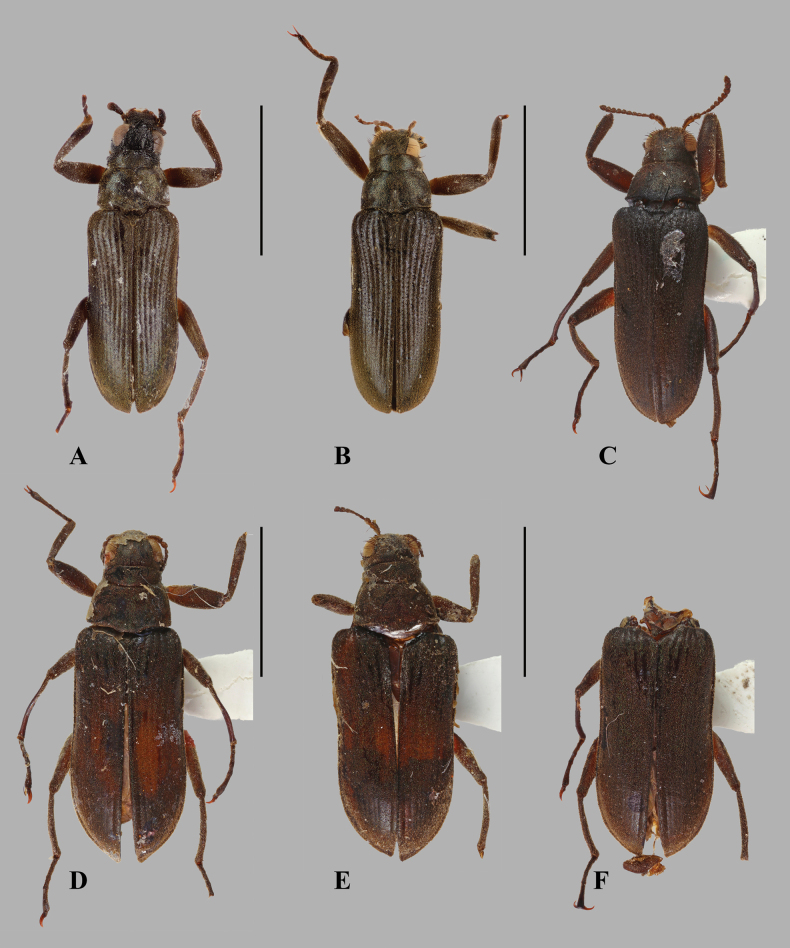
Dorsal habiti of *Hexanchorus tibialis* Hinton, 1935. **A**. Male, holotype [NHMUK]; **B**. Male, non-type [MNHN]; **C**. Male, non-type [MNHN]; **D**. Female, non-type [MNHN]; **E**. Female, non-type [MNHN]; **F**. Female, non-type [MNHN]. Photographs **C–F** taken by Christophe Rivier (MNHN). Scale bars: 2 mm.

The holotype (Fig. 1A) lacks antennae; the left protarsus lacks the last two segments, the right protarsus has only the first segment intact; middle legs are missing; the left metatarsus is incomplete, lacking the last two segments. The last two abdominal ventrites, as well as the sixth ventrite, are glued next to the specimen.

##### Non-type material examined.

(2 ♂♂, 3 ♀♀) Bolivia • ♂ “♂// Yungas de Cochabamba Bolivie // Type // Xexanchorus montanus = ined. Grouv // J. Delève det., 1967 Hexanchorus tibialis Hntn // Muséum Paris // Hexanchorus tibialis Hinton, 1935 Vidit Thiago Polizei 2017” (MNHN) • 1♂, 3♀♀ [examined from photographs only] sharing the following three labels: “Yungas de Cochabamba Bolivie // Museum Paris Coll. A. Grouvelle 1917 // J. Delève det., 1966 Hexanchorus tibialis Hntn”, differing only in the fourth label “Muséum Paris” followed by accession numbers EC56316 (♂) and EC56313–EC56315 (♀♀), (MNHN).

##### Diagnosis.

Males of *Hexanchorus tibialis* can be distinguished from all species of the genus by combination of the following characters: 1) large size, CL 3.52–3.63 mm (*n* = 3); 2) mesotibia with anterior lateral pubescent area in basal 1/5, posterior lateral pubescent area long at basal 1/2; 3) mesotibia with a distinct apical tubercle on the inner side; 4) metatibia with a small and obliquely longitudinal apical tubercle on the inner side; 5) elytral apices rounded; 6) penis gradually narrowing towards apex from ca basal 1/3, subapically abruptly constricted into a rounded apex.

##### Redescription.

**Male**. Body elongate, subparallel, dorsum moderately convex (Fig. 1A–C). CL 3.52–3.63 mm (*n* = 3); EW 1.24–1.28 mm (*n* = 3). Dorsal surface densely covered with fine, short, recumbent, grey setae, often with green iridescence, and with sparser, longer, darker, semierect setae. *Colour*: cuticle black to dark reddish brown; basal antennal segments I–II, mouthparts, trochanters, bases of femora, mesotibiae, and claws paler.

***Head*** partly retractable into prothorax, without distinct impressions. Surface microreticulate, with dense, fine punctures and sparse, coarse punctures. Clypeus with anterior margin truncate, anterior angles broadly rounded. Labrum feebly emarginate anteromedially, expanded laterally with sides broadly rounded, densely setose. Eyes suboval in lateral view, protruding from head outline, bordered by long, dark, curved setae (“eyelashes”) that arise near dorsal and ventral sides of eyes and extend toward the middle of the eye. Antennae 11-segmented, densely pubescent with short, pale setae, first two segments also with numerous long, darker, hair-like setae.

***Thorax***. Pronotum PL 0.76–0.82 mm (*n* = 3), PW 0.94–0.95 mm (*n* = 3), widest at basal 2/5; with complete transverse depression in apical third, small basolateral impressions, and two prescutellar foveae; sublateral carinae absent; medial sulcus indistinct; lateral margins convex before and after depression; anterior margin arcuate, anterior angles rounded; posterior margin trisinuate, broadly arcuate on each side and narrowly in front of scutellum; posterior angles suborthogonal. Scutellum subtriangular. Hypomera moderately wide, narrowed in the middle. Legs slender and very long. Pro- and metatibia fully clothed; mesotibia with anterior lateral pubescent area in basal 1/5, posterior lateral pubescent area long at basal 1/2. Apex of mesotibia with a distinct tubercle on inner side, apex of metatibia with a small and obliquely longitudinal tubercle. Protarsus simple (Fig. 6A). Fourth tarsomere with a distinctly longer, apicoventral, erect, hair-like seta about half as long as the last tarsal segment. Tarsal claws long and stout. Elytra EL 2.70–2.87 mm (n = 3), EW 1.24–1.28 mm (n = 3), widest across humeri; more than three times longer than pronotum, subparallel in anterior 4/5. Each elytron with 10 rows of small punctures; punctures on the mid-disc round, separated longitudinally by 2–4 times their diameter, becoming finer and shallower toward sides and apex, and nearly effaced apically. Intervals flat to slightly depressed; sutural interval weakly raised on the posterior half. Lateral margins smooth; apex narrowly and evenly rounded on the inner side. Epipleura narrow, oblique near basal 1/3, then inflexed horizontally.

**Figure 2.**
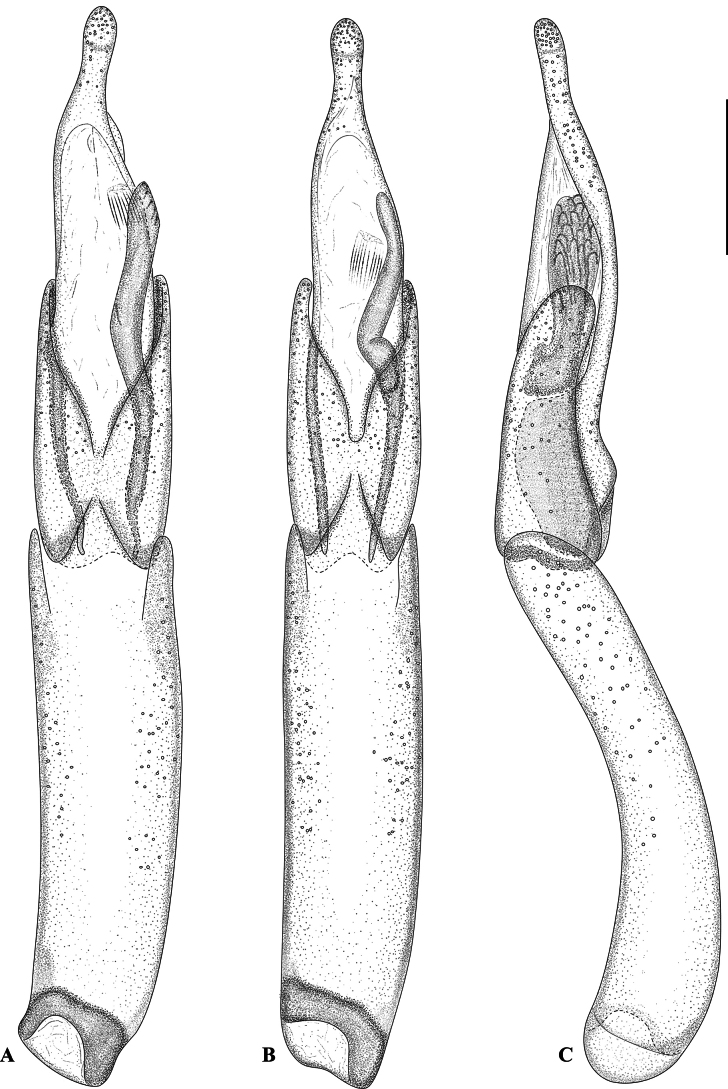
Aedeagus of *Hexanchorus tibialis* Hinton, 1935. **A**. Ventral view, non-type [MNHN]; **B**. Ventral view, holotype [NHMUK]; **C**. Lateral view, holotype [NHMUK]. Scale bar: 0.1 mm.

***Aedeagus*** (Fig. 2) elongate and slender, penis almost subparallel, apically gradually narrowing from approximately basal 1/3, subapically abruptly constricted into a rounded apex. Corona membranous, dorsal fibula absent. Membranous sack with a long, slender, weakly sinuate ventral sclerite (fibula major sensu Linský et al. 2022). Parameres about half as long as penis, in ventral view fused in the middle, in lateral view widest at base, tapering towards broadly rounded apex. Phallobase slightly longer than penis, in ventral view subparallel, in lateral view curved. Sternite VIII (Fig. 5D) with long and stout median strut; apically broadly bisinuate; densely set with moderately long, curved, semierect, hair-like setae.

**Female** externally similar to male, slightly larger; elytral apex produced with inner margin nearly straight; meso- and metatibia without apical tubercle on inner side (Figs 1D–F). Females vary in size (CL 3.95–4.04 mm [*n* = 3], PL 0.86–0.91 mm [*n* = 2], PW 1.06–1.09 mm [*n* = 2], EL 3.04–3.16 mm [*n* = 3], EW 1.44–1.51 mm [*n* = 3]).

##### Remarks.

Ventral side and female genitalia were not examined.

##### Distribution.

Known from the Yungas of Cochabamba, Bolivia (for possible collecting sites, see Discussion), and Cusco and Madre de Dios regions, Peru (Shepard and Chaboo 2015; W.D. Shepard pers. comm.).

##### Comparative notes.

This species differs from its congener *H. siriono* sp. nov. by the absence of laterally dilated protarsi in males, by the shorter anterior (basal 1/5 vs basal 1/3) and longer posterior (basal 1/2 vs basal 1/3) lateral pubescence on the mesotibia, and by the median lobe, which is apically constricted (vs apically expanded laterally in *H. siriono* sp. nov.). The lack of sexual dimorphism in the protarsi and profemora, together with a more distinct mesotibial apical carina and a less distinct metatibial one, places this species near several Ecuadorian *Hexanchorus* species. Most of these differ from *H. tibialis* by a greater extent of mesotibial pubescence (anteriorly reaching at least the midlength vs basal 1/5 and posteriorly reaching at least basal 2/3 vs basal 1/2), except *H. rostratus* Linský et al., 2019, in which it is restricted to the extreme base anteriorly and to the basal 1/4 posteriorly. These two species differ in the extent of mesotibial pubescence, in the shape of the elytral apices (rounded vs feebly produced and projected outwards), and in the morphology of the median lobe, which is wider with a preapical constriction in *H. tibialis*, and narrow, gradually tapering into an apex in *H. rostratus* (Linský et al. 2019; unpublished data).

#### Hexanchorus siriono sp. nov.

Taxon classificationAnimaliaColeopteraElmidae

670EFF45-6C69-55AD-A865-0E977D9F497F

https://zoobank.org/7703C48F-D775-4B26-907E-0E1B05D61880

**Figure 3.**
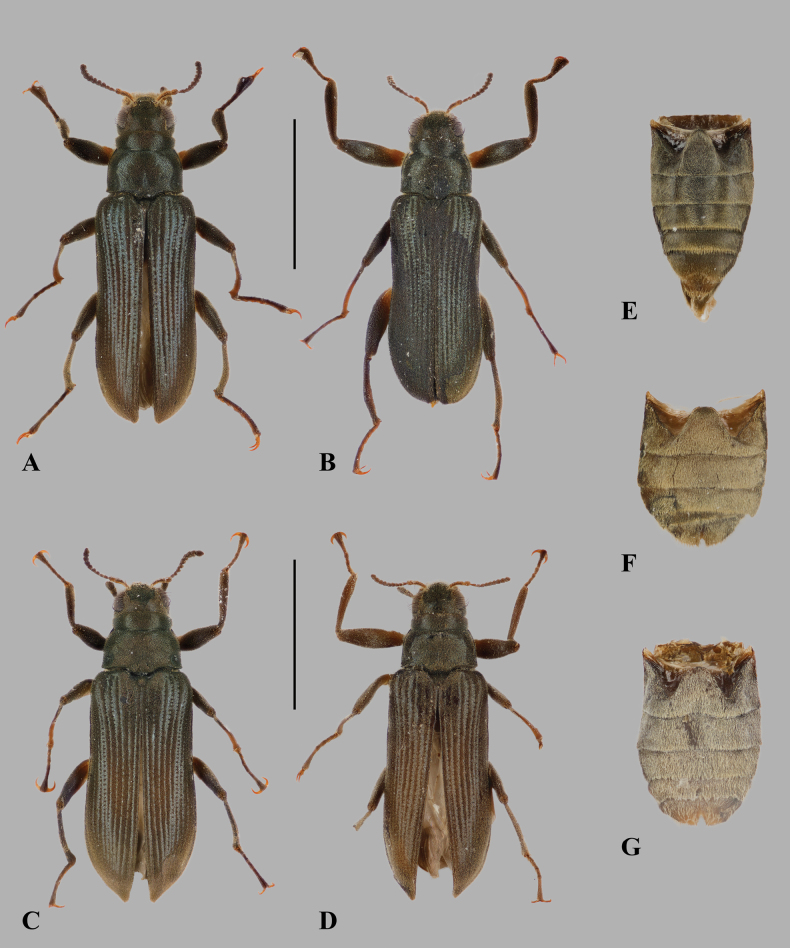
Dorsal habiti and abdomens of *Hexanchorus siriono* sp. nov. **A**. Male, holotype [NHMUK]; **B**. Male, paratype [NHMUK]; **C**. Female, allotype [NHMUK]; **D**. Female, paratype [OMNH]; **E**. Male abdomen, holotype [NHMUK]; **F**. Female abdomen, allotype [NHMUK]; **G**. Female abdomen, paratype [OMNH]. Scale bars: 2 mm (A–D); E–G not to scale.

**Figure 4.**
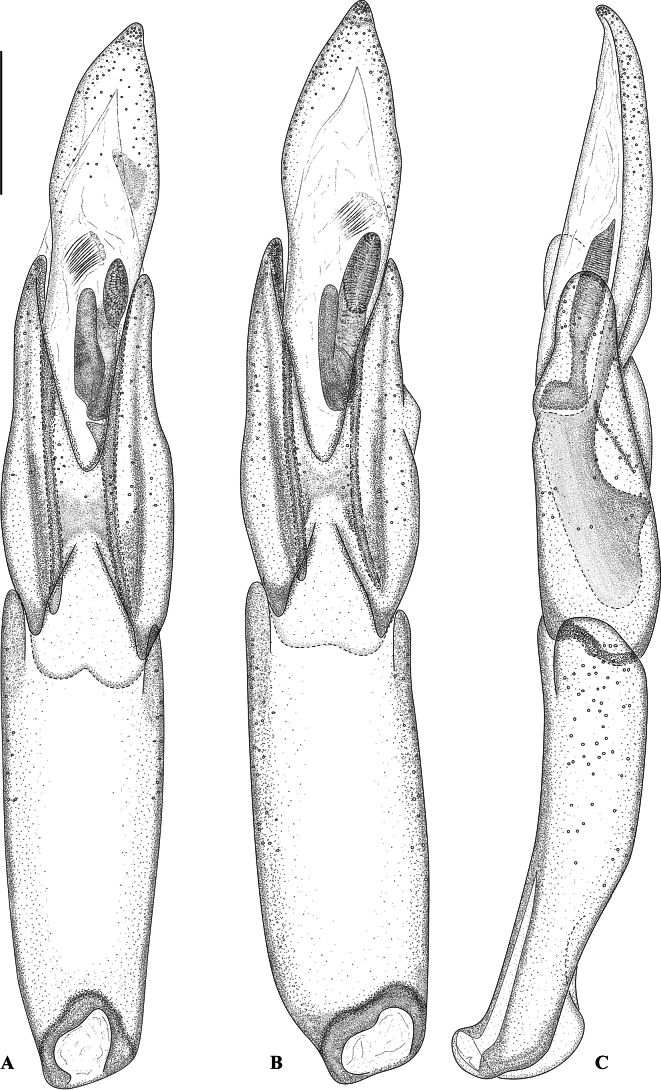
Aedeagus of *Hexanchorus siriono* sp. nov. **A**. Ventral view, paratype [NHMUK]; **B**. Ventral view, holotype [NHMUK]; **C**. Lateral view, holotype [NHMUK]. Scale bar: 0.1 mm.

**Figure 5.**
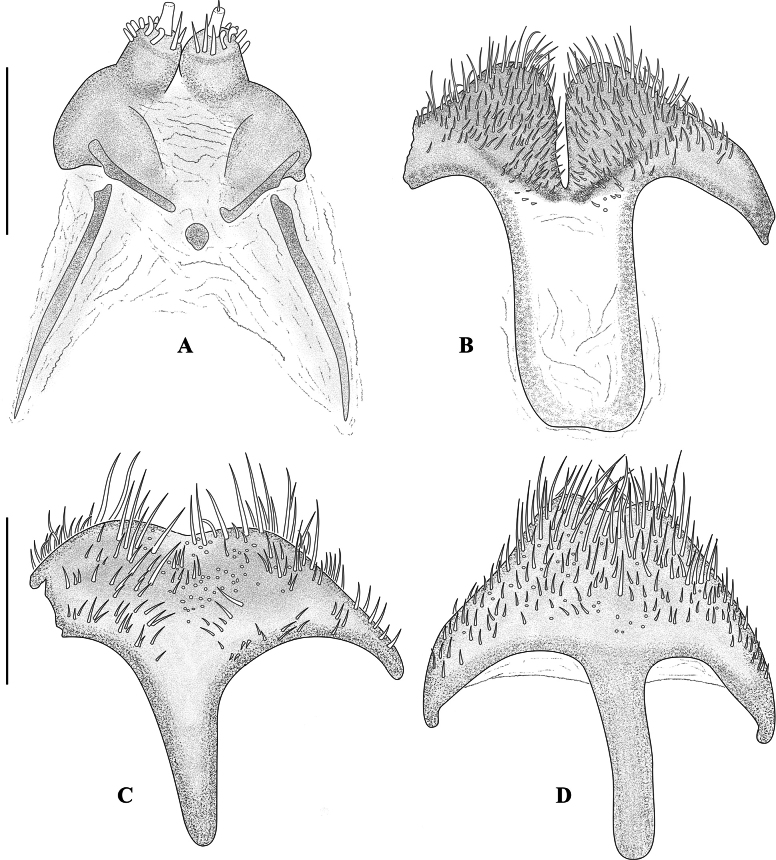
Genital structures of *Hexanchorus siriono* sp. nov. and *H. tibialis* Hinton, 1935. **A**. Ovipositor, paratype [OMNH]; **B**. Female sternite VIII, paratype [OMNH]; **C**. Male sternite VIII, paratype [NHMUK]; **D**. Male sternite VIII of *H. tibialis*, holotype [NHMUK]. Scale bars: 0.1 mm.

**Figure 6.**
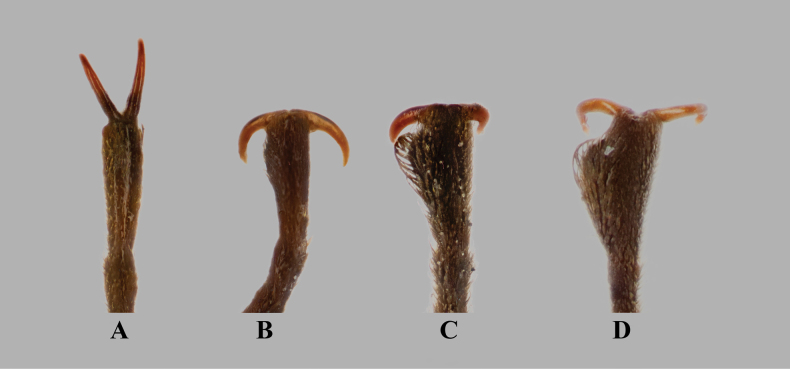
Male protarsi of selected *Hexanchorus* species. **A**. *H. tibialis* Hinton, 1935, non-type [MNHN]; **B**. *H. caraibus* (Coquerel, 1851), non-type [OMNH]; **C**. *H. tarsalis* Hinton, 1937, holotype [NHMUK]; **D**. *H. siriono* sp. nov., paratype [NHMUK]. Not to scale.

**Figure 7.**
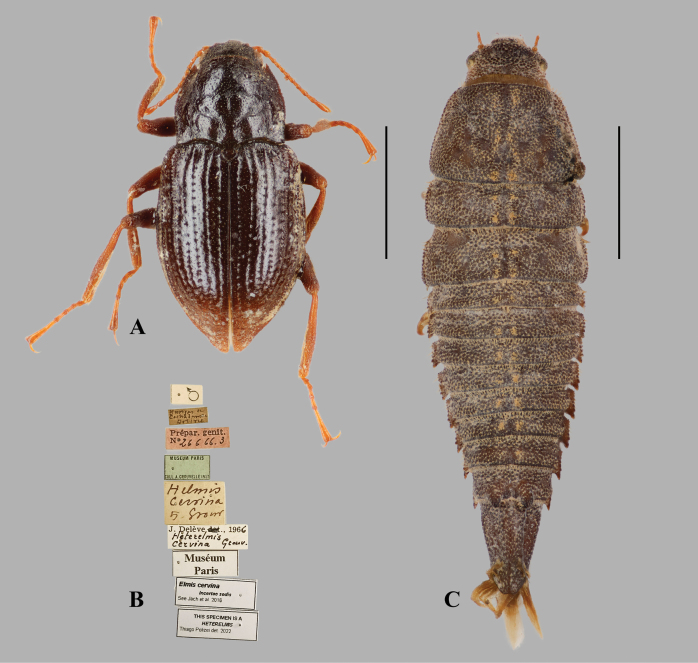
*Heterelmis cervina* (Grouvelle, 1896), comb. nov. and *Hexanchorus* larva. **A**. Dorsal habitus of *H. cervina*, male [MNHN]; **B**. Labels of the same specimen; **C**. *Hexanchorus* larva collected with *H. siriono* sp. nov. [OMNH]. Scale bars: 1 mm (**A**), 2 mm (**C**); (**B**) not to scale.

Figs 3, 4, 5 A–C, 6D, 7C

##### Type material.

(5♂♂, 3♀♀) ***Holotype***: Bolivia • ♂ “♂ // BOLIVIA: Santa Cruz Amboro National Park Los Volcanes, c.1000m 18° 06'S, 63° 36'W 20/xi-12/xii/2004 // MV Light Sheet by tree fall Barclay, M.V.L. & Mendel, H. BMHN(E)2004-280 // Hexanchorus tibialis Hinton, 1935 T.T.S.Polizei det. 2017” (NHMUK). ***Allotype***: • ♀ as in the holotype, except for the female gender symbol (NHMUK). ***Paratypes***: • 4♂♂ as in the holotype (gender symbol missing in one specimen), (NHMUK); • 2♀♀ “BOLIVIA: Santa Cruz Rio la Negra (mts.) 71/11/8 H.P.Brown // Catalog No. OMNH-112373 // Hexanchorus det. H.P.B.” (OMNH).

##### Putative additional material.

Bolivia • 2 larvae “BOLIVIA: Santa Cruz Rio la Negra (mts.) 71/11/8 H.P.Brown // Catalog No. OMNH-112373 // Hexanchorus det. H.P.B.” (OMNH).

##### Remarks.

Two larvae (Fig. 7C) were collected together with two females at the Río La Negra locality. They belong to the genus *Hexanchorus*, as they have a fusiform, slightly dorsoventrally depressed body with setose lateral thoracic and abdominal extensions, a rounded terminal segment, a dorsal tubercle on each side of the posterior margin of segment VIII, and ventral pleural sclerites on abdominal segments I–VI (Manzo and Archangelsky 2008; Linský et al. 2022). They may or may not belong to *H. siriono* sp. nov. because species-level diagnostic larval characters for *Hexanchorus* have not been documented so far.

##### Diagnosis.

Males of *Hexanchorus siriono* sp. nov. can be distinguished from all species of the genus by combination of the following characters: 1) large size CL 3.56–3.90 mm (*n* = 5); 2) protarsi strongly dilated laterally; 3) mesotibia with anterior lateral pubescent area reaching slightly behind basal 1/3, posterior lateral pubescent area in basal 1/3; 4) mesotibia with a distinct tubercle on the inner side of tibial apex; 5) metatibia with a distinct tubercle on the inner side of tibial apex; 6) elytral apices with inner margins obliquely truncate; 7) fifth ventrite apically moderately deeply and broadly emarginate; 8) penis subparallel in basal 3/5, apically expanded into a blunt, arrowhead-like apex.

##### Description.

**Male**. Body elongate, subparallel (Fig. 3A, B), dorsum moderately convex. CL 3.56–3.90 mm (*n* = 5), EW 1.27–1.37 mm (*n* = 5). Dorsal surface densely covered with fine, short, recumbent, grey setae, often with green iridescence, and with sparser, longer, darker, semierect setae. Ventral surface densely clothed with fine, long, golden, recumbent setae forming a tightly interlaced layer, together with longer and slightly thicker golden, semierect setae. ***Colour***: cuticle black to dark reddish brown; basal antennal segments I–II, mouthparts, trochanters, bases of femora, mesotibiae, and claws paler.

***Head*** partly retractable into prothorax, without distinct impressions. Surface microreticulate, with dense, fine punctures and sparse, coarse punctures. Clypeus with anterior margin truncate, anterior angles broadly rounded. Labrum densely setose, feebly emarginate anteromedially, expanded laterally with sides broadly rounded. Eyes suboval in lateral view, protruding from head outline, bordered by long, dark, curved setae (“eyelashes”) that arise near dorsal and ventral sides of eyes and extend toward the middle of the eye. Antennae eleven segmented, densely pubescent with short, pale setae, first two segments also with numerous long, darker, hair-like setae.

***Thorax***. Pronotum PL 0.82–0.86 mm (*n* = 5), PW 0.95–1.02 mm (*n* = 5), widest at basal 2/5; with complete transverse depression in apical third, small basolateral impressions, and two prescutellar foveae; sublateral carinae absent; with feeble medial sulcus in basal half; lateral margins convex before and after depression; anterior margin arcuate, anterior angles rounded; posterior margin trisinuate, broadly arcuate on each side and narrowly in front of scutellum; posterior angles suborthogonal. Scutellum subtriangular. Hypomera moderately wide, narrowed in the middle. Prosternum very short in front of procoxae; prosternal process elongate, broad at base, tapering apically to a subacute apex. Mesoventrite short, with a broad, deep, median depression for reception of the prosternal process. Metaventrite long and wide, disc broadly concave with a narrow longitudinal impression on midline; anterior margin concave laterally and convex medially. Legs slender and very long. Pro- and metatibia fully clothed; mesotibia with anterior lateral pubescent area reaching slightly behind basal 1/3, posterior lateral pubescent area in basal 1/3. Apices of meso- and metatibia each with a distinct tubercle on inner side. Protarsus strongly dilated on inner half (Fig. 6D). Fourth tarsomere with a distinctive, apicoventral, long, erect, hair-like seta about half as long as the last tarsal segment. Tarsal claws long and stout. Elytra EL 2.75–3.04 mm (*n* = 5), EW 1.27–1.37 mm (*n* = 5), widest across humeri; more than three times longer than pronotum, subparallel in anterior 4/5. Each elytron with 10 rows of small punctures; punctures on the mid-disc round, separated longitudinally by 2–4 times their diameter, becoming finer and shallower toward the sides and apex, and nearly effaced apically. Intervals flat to slightly depressed; sutural interval weakly raised on the posterior half. Lateral margins smooth; apex with inner margin obliquely truncate. Epipleura narrow, oblique near basal 1/3, then inflexed horizontally.

***Abdomen*** (Fig. 3E) with five clearly visible ventrites. Intercoxal process subtriangular with rounded apex. First three ventrites concave medially; fifth ventrite apically moderately deeply and broadly emarginate. Cuticle densely covered with short, golden, recumbent setae. ***Aedeagus*** (Fig. 4) robust but elongate, penis subparallel in basal 3/5, apically expanded into a blunt, arrowhead-like apex. Corona membranous, dorsal fibula absent. Membranous sac with a large, apically narrowed ventral sclerite (fibula major). Parameres about 2/3 as long as penis, in ventral view fused in the middle, in lateral view widest at base, tapering towards broadly rounded apex. Phallobase slightly shorter than penis, in ventral view subparallel, in lateral view curved. Sternite VIII (Fig. 5C) with long and stout median strut; apically broadly bisinuate; set with moderately long, curved, semi-erect, hair-like setae.

**Female**. (Fig. 3C, D) externally similar to male, slightly larger; elytral apex produced with inner margin obliquely truncate; meso- and metatibia without apical tubercle on inner side; protarsus simple; first three ventrites medially convex and fifth ventrite apically with deep, but moderately narrow, arcuate emargination (Fig. 3F, G). ***Ovipositor*** (Fig. 5A) with coxites and styli short; coxites broad at base; baculi long and slender. Sternite VIII (Fig. 5B) with long and rather wide median strut; apically bisinuate, very deeply but narrowly emarginate; densely set with moderately long, curved, semi-erect, hair-like setae. Females vary in size (CL 3.81–3.90 mm, PL 0.77–0.85 mm, PW 0.97–1.04 mm, EL 2.98–3.07 mm, EW 1.34–1.42 mm; *n* = 3).

##### Variation.

The holotype (CL 3.90 mm, PL 0.86 mm, PW 1.02 mm, EL 3.04 mm, EW 1.37 mm; *n* = 1) is larger than the other four males (CL 3.56–3.66 mm, PL 0.82–0.83 mm, PW 0.95–0.96 mm, EL 2.75–2.84 mm, EW 1.34–1.42 mm; *n* = 4), but they agree in all other characters, including body shape, structure of the aedeagus, male secondary sexual characters, mesotibial pubescence, and the shape of the elytral apices.

##### Etymology.

The Siriono, are indigenous people of eastern Bolivia who live a semi-nomadic foraging life with minimal material culture. They are traditionally known for their use of exceptionally long bows, 2–3 m in length, with even longer arrows, which they employ in subsistence hunting (Holmberg 1969). The name, a noun in apposition, refers to the geographic connection between the Siriono homeland and the type locality (Santa Cruz, eastern Bolivia), and to the arrowhead-like apex of the aedeagus. The group is also vividly portrayed by Germain in his travel writings on Bolivia.

##### Distribution.

Known from two localities in the Santa Cruz Department, Bolivia: one in Los Volcanes, Amboró National Park, and the other from “Río La Negra”. The latter likely refers to a stream near the settlement of La Negra (Mapcarta 2025), possibly the arroyo (quebrada) La Negra that flows into the Río Laja, where the river changes its name to Río Bermejo. If so, both localities are less than 10 km apart.

##### Comparative notes.

This species resembles *H. caraibus* (Coquerel, 1851) and *H. tarsalis* Hinton, 1937 in having the protarsi laterally dilated in males, but differs from both by its larger body size (CL 3.56–3.90 mm in *H. siriono* sp. nov., 2.82–3.16 mm in *H. caraibus*, 3.28–3.45 mm in *H. tarsalis*), the presence of distinct carinae ending in a single tubercle on the inner margins of the meso- and metatibial apices (vs only feeble, longitudinal carinae in the other two species), and by a greater extent of lateral pubescence on the mesotibia (restricted to the extreme base in *H. caraibus* and to the basal 1/6 in *H. tarsalis*). The median lobe of *H. siriono* sp. nov. is subparallel in the basal 3/5, then apically expanded into a blunt, arrowhead-like apex, whereas in *H. caraibus* and *H. tarsalis* it is subparallel and straight basally, then gradually narrowing to a rounded apex without lateral expansion (Spangler and Santiago-Fragoso 1992; unpublished data).

#### Heterelmis cervina

Taxon classificationAnimaliaColeopteraElmidae

(Grouvelle, 1896) comb. nov.

BC01D0D2-F6F4-522C-9C49-741043E6887B

Fig. 7A, B

Helmis cervina Grouvelle, 1896: 51.

##### Type material examined.

***Lectotype*** (here designated): Bolivia • ♂ “♂ // Yungas de Cochabamba Bolivie // Prépar. Genit. N 26666.3 // Museum Paris Coll. A.Grouvelle 1917 // Helmis cervina ty. Grouv [according to A. Mantilleri (pers. comm.), the abbreviation “ty.” was used by Grouvelle to indicate specimens belonging to the type series] // J. Delève 1966 Heterelmis cervina Grouv. // Muséum Paris // Elmis cervina incertae sedis See Jäch et al. 2016 // This specimen is a Heterelmis Thiago Polizei det. 2022” (MNHN).

##### Remarks.

In the original description, Grouvelle (1896) stated that the type was in his collection but did not indicate how many specimens were examined. Only a single specimen attributable to this taxon was found in Grouvelle’s collection (A. Mantilleri pers. comm.). This specimen clearly belongs to *Heterelmis*, as it has a subparallel body, a pronotum with sublateral carinae extending from the base almost to the anterior margin, a transverse median impression, a short median longitudinal groove, an oblique impression on each side of the basal half, and elytra with longitudinal carinae on intervals VI and VIII (Hinton 1940a). Since it is unclear whether the type series originally consisted of a single specimen (in which case it would constitute a holotype by monotypy), we adopt a more cautious approach and treat it as a syntype, which we hereby designate as lectotype. This species raises the number of known species of this genus in Bolivia to three, together with *H. longior* (Grouvelle, 1896) and *H. neglecta* Grouvelle, 1896.

## Discussion

### Germain’s travels

The itinerary of Germain’s journeys has never been published, but much about his life and travels can be inferred from his own publications. Philibert Germain (January 25, 1827 – December 9, 1913) was a French-born naturalist who left his country for Chile in 1850, where he collected insects and birds that he prepared as taxidermy specimens. Shortly afterward, he was offered a position at the Chilean National Museum of Natural History and worked there for five years. After his resignation, he returned to Europe, where he spent nearly two decades (Germain 1910). Driven by his old passion for natural history and entomology, he once again crossed the ocean in 1881 and arrived in Brazil, where he spent several years collecting insects that he sent to L. Fairmaire in Paris and R. Oberthür in Rennes (Germain 1898, 1910).

From Brazil, Germain travelled to Paraguay, preparing for a long entomological exploration of Bolivia. On August 22, 1887, he sailed from Asunción to Corumbá, Brazil (Germain 1898). He reached Corumbá on September 5 (Germain 1899) and, after spending a few days there, left on September 17, crossing into Bolivia and arriving in Santa Cruz de la Sierra on October 13 (Germain 1900a). He spent 10 months in the region, collecting extensive and scientifically valuable material, mainly Lepidoptera, although the entire collection was later lost or stolen during its transport to Europe (Germain 1900b).

Germain’s published accounts provide little information about his activities during the latter part of 1888, but he began the year 1889 by stating that his exploration of the Yungas of the Espíritu Santo River had been completed and that he was preparing for a journey toward La Paz. The plan then took an unexpected turn when he met a young Englishman in Cochabamba who was managing a large estate in the valleys formed by the headwaters of the Beni River, and Germain decided to explore this region instead. The expedition was postponed until the end of February, when they finally left Cochabamba. Over the following months, Germain described numerous obstacles encountered while crossing the mountain range, followed by a descent into the Yungas, which they finally reached on June 15, when the party arrived at the right bank of the Santa Elena valley. They encamped at Cuesta-cique, where they remained until August, during which time Germain enriched his insect collections (Germain 1897).

On August 15, Germain set out on foot for Charoplaya, a camp 30 km away, crossing the Santa Elena River twice before reaching the site after eight hours of travel. He remained there for two months, collecting many beetles and butterflies. In early October, the rainy season began unexpectedly, and within a few days the level of the Santa Elena had risen significantly, which made further stay in the area dangerous. Consequently, his companions decided to withdraw, and on October 12 Germain left the Yungas, retracing his route through San Carlos and Choro, finally reaching Cochabamba on November 24 (Germain 1897).

All examined riffle-beetle specimens collected by Philibert Germain from Bolivia and housed in the MNHN collection that still bear his original handwritten labels originate from the Yungas of Cochabamba (e.g. the second label from the top in Fig. 7B). Numerous other Bolivian specimens attributed to Germain bear printed labels reading “Cochabamba” but lack the locality detail present in his handwriting. It is therefore plausible that the printed labels correspond to that same region, although the explicit locality information was lost during later relabelling. Details about his exploration of the Yungas of the Espíritu Santo River are very scarce. If these Bolivian specimens are not from that region, they were most likely collected in the Santa Elena valley between June 15 and October 12, 1889.

### Bolivian *Hexanchorus*

Hinton (1935) described *Hexanchorus tibialis* from a single male specimen from the Grouvelle collection, originally collected by Germain in the Yungas of Cochabamba. The holotype, deposited in the NHMUK, is in poor condition, lacking antennae, middle legs, and both protarsi. The species was diagnosed mainly by the presence of a fine carina forming a small apical tubercle on the inner side of the hind tibiae. However, such a fine carina on the tibial apex, often shaped as a small tubercle, is present in all *Hexanchorus* species except *H. angeli* Laššová et al., 2014 (and possibly other species from the Guiana Shield; Linský et al. 2022).

Due to the poor preservation of the holotype, recently collected Bolivian material was misidentified as *H. tibialis*. The males of these specimens possess conspicuously dilated protarsi—a character that could not be verified in the type specimen. Examination of this material, including male genitalia and other morphological features, revealed that it represents a distinct species, herein described as *Hexanchorus siriono* sp. nov.

Additional material housed in the MNHN, collected from the same locality as the holotype, corresponds to *H. tibialis* and confirms that males of this species have simple protarsi (Fig. 6A). Our observations also corroborate the findings of Linský et al. (2022), who noted that males and females of *Hexanchorus* exhibit the same extent of pubescence on the mesotibia—a feature that can be reliably used to associate the sexes.

Dilated protarsi were first described in *H. tarsalis* Hinton, 1937 from Brazil (Hinton 1937) and later reported in *H. caraibus* (Coquerel, 1851) from the Lesser Antilles (Spangler and Santiago-Fragoso 1992). *Hexanchorus siriono* sp. nov. therefore represents the third known species in the genus exhibiting this distinct male sexual dimorphism (for comparison, see Fig. 6B–D). In most *Hexanchorus* species, females are generally larger than males. For *H. siriono*, this pattern holds true when the male holotype (reaching 3.90 mm) is excluded, as the remaining males measure 3.56–3.66 mm and females 3.81–3.90 mm. The reason for this deviation is unclear, as the holotype agrees in all other morphological characters with the remaining males (for comparison, see Figs 3A, 3Band 4A, B). It could be caused by individual variation, potentially influenced by larval nutrition, or by population-level divergence with limited dispersal. However, resolving this question is not currently possible due to the small sample size.

The description of *H. siriono* sp. nov. increases the number of *Hexanchorus* species known from Bolivia to two and brings the total number of described species in the genus to 26. The detailed redescription of *H. tibialis*, based on both the type and additional topotypic material, further improves our understanding of the genus. Although most known *Hexanchorus* species are now adequately characterized, with the sole exception of *H. thermarius* (Coquerel, 1851), the genus is still far from completely understood, and numerous undescribed species are recognized but await formal description.

## Supplementary Material

XML Treatment for Hexanchorus tibialis

XML Treatment for Hexanchorus siriono

XML Treatment for Heterelmis cervina

## Additional information

### Use of AI

No use of AI was reported.

### Author ORCIDs

Marek Linský https://orcid.org/0000-0001-7206-2380

Fedor Čiampor https://orcid.org/0000-0001-6269-3592

### Data availability

All of the data that support the findings of this study are available in the main text.
